# An Instrumental Measure of Hand and Facial Movement Abnormalities in Patients With Schizophrenia

**DOI:** 10.3389/fpsyt.2022.803661

**Published:** 2022-03-04

**Authors:** Shu-Mei Wang, Wen-Chen Ouyang, Hsiao-Man Hsu, Li-Ta Hsu

**Affiliations:** ^1^Department of Rehabilitation Sciences, The Hong Kong Polytechnic University, Kowloon, Hong Kong SAR, China; ^2^Department of Geriatric Psychiatry, Jianan Psychiatric Center, Ministry of Health and Welfare, Tainan, Taiwan; ^3^Department of Nursing, Shu-Zen Junior College of Medicine and Management, Kaohsiung, Taiwan; ^4^Department of Psychiatry, College of Medicine, Kaohsiung Medical University, Kaohsiung, Taiwan; ^5^Institute of Biomedical Engineering, College of Engineering, National Cheng Kung University, Tainan, Taiwan; ^6^Department of Aeronautical and Aviation Engineering, The Hong Kong Polytechnic University, Kowloon, Hong Kong SAR, China

**Keywords:** bradykinesia, dyskinesia, hand, face, schizophrenia

## Abstract

**Introduction:**

Movement disorders have been suggested to be a cardinal component of schizophrenia. With increased research interests in this area, instrumental measures are needed. This study was to examine if the motion capture system was reliable in measuring hand and facial bradykinesia and dyskinesia and more sensitive to detecting movement differences between schizophrenia patients and healthy people than traditional rating scales.

**Methods:**

Sixteen schizophrenia patients and 20 control subjects were recruited. Hand and facial bradykinesia and dyskinesia were measured using the motion capture system and rated using the Extrapyramidal Symptom Rating Scale and the Abnormal Involuntary Movement Scale.

**Results:**

The system showed strong test–retest reliability and generated larger effect sizes of group differences than did the rating scales.

**Conclusions:**

The results may support researchers and clinical practitioners to apply the system to sensitively measuring the hand and facial movement symptoms in schizophrenia patients, which contributes to gaining a deep understanding of movement issues in schizophrenia.

## Introduction

Movement disorders have been suggested to be a cardinal component of schizophrenia (1–3). Aberrant movements, such as bradykinesia (slow movements) and dyskinesia (involving irregular and involuntary muscle contraction), at any body parts are present across different stages of schizophrenia, including the at-risk stage (4–6), the first-episode drug-naive stage (2), and the chronic stage (7). In addition, evidence has indicated that the movement abnormalities are tied to altered basal ganglia (4, 5), which are known to play a pivotal role in the etiology of schizophrenia (8, 9). Even though movement symptoms in schizophrenia have gained increasing research attention (10, 11), limitations of traditional movement assessment strategies may hamper research development in this area. Movement problems in schizophrenia are usually assessed using clinical observation–based rating scales (10, 11), such as the Extrapyramidal Symptom Rating Scale (ESRS) for assessing bradykinesia and the Abnormal Involuntary Movement Scale (AIMS) for assessing dyskinesia. However, these scales suffer from limitations of being vulnerable to rater bias and being insensitive to minimal to mild movement differences and the requirement of intensive training to secure reliability (1, 10–13). With increased interests in exploring movement issues in schizophrenia, researchers have emphasized that objective and sensitive instrumental measures are undoubtedly needed (1, 10–13).

Previous pioneering studies have developed mechanical measures (e.g., handwriting analysis via Wacom digitizing tablets; strain gauges; load cells; electrogoniometers) to detect bradykinesia and dyskinesia in schizophrenia patients or their siblings (12, 14–19). However, the developed instruments mainly target hand movement problems. Measurement of aberrant movements at other body parts, such as the face, is still unable to benefit. In addition, the use of different apparatus to measure bradykinesia and dyskinesia reduces convenience of examining varying aberrant movements in patients. For the past decades, motion analysis through using commercial motion capture systems has been extensively applied to sensitively detecting multiple movement problems at different body parts and measuring movement improvements after treatment in patients with neurological dysfunction such as stroke (20, 21) and Parkinson disease (22–24). By recording movement trajectories of markers/sensors attached to the patient's body parts, motion capture systems make it possible to calculate kinematic variables that objectively and directly reflect movement speed and quality for each body part in the patient. Existing studies have applied motion capture systems to measuring hand movements in patients with schizophrenia (25–27). Nevertheless, to date, little has been known about whether motion capture systems reliably measured hand and facial bradykinesia and dyskinesia in schizophrenia patients. Moreover, it remains uncertain whether motion capture systems were more sensitive to detecting differences in hand and facial bradykinesia and dyskinesia between schizophrenia patients and healthy people than clinical rating scales.

To sum up, this study aimed to examine (1) if the motion capture system was reliable in measuring hand and facial bradykinesia and dyskinesia and (2) if the system was more sensitive to detecting differences in hand and facial bradykinesia and dyskinesia between schizophrenia patients and healthy people than the clinical rating scales. We hypothesized that (1) the motion capture system had strong test–retest reliability for measuring hand and facial bradykinesia and dyskinesia, and (2) the effect sizes of group differences in hand and facial bradykinesia and dyskinesia measured using the system were larger than those of group differences in hand and facial bradykinesia and dyskinesia assessed using the clinical rating scales. This study provides a measuring procedure of the motion capture system. The results will support application of the motion capture system to movement studies in schizophrenia and thus further benefit an in-depth understanding of movement issues in psychotic patients.

## Methods

### Participants

Schizophrenia patients were recruited from the outpatient clinic of a hospital and community self-help groups. The inclusion criteria for patients were a diagnosis of schizophrenia without other psychiatric diagnoses confirmed by psychiatrists according to the *Diagnostic and Statistical Manual of Mental Disorders, Fifth Edition* (28), a score of 22 or greater on the Montreal Cognitive Assessment (29) to show that patients were able to understand experimental instructions, and a score >60 on the Edinburgh Handedness Inventory (30) to show right-handedness. Healthy people were recruited from communities and met the previously mentioned inclusion criteria concerning the Montreal Cognitive Assessment and the Edinburgh Handedness Inventory. Exclusion criteria for participants were presence of medical conditions or neurological diseases that affected hand movements or facial expression. This study has been reviewed by the ethical review boards of the university (reference no. HSEARS20190322003) and the hospital (reference no. KC/KE-18-0118/FR-2). Consent forms signed by participants were obtained before the study.

### Clinical Observation–Based Rating Scales

#### For Assessing Bradykinesia: ESRS

The ESRS is a clinical measure for assessing movement disorders in schizophrenia patients (31). The hypokinesia factor of ESRS includes seven items: rigidity for four limbs, expressive automatic movement disorders (facial mask and unintelligible speech), slowness at movement initiation, and gait and posture abnormalities (31). The score of each item ranges from zero (none) to six (most severe). In order to prevent multiple testing and the subsequent inflated type I error rate, this study chose to analyze movements of the right hand and the right face (the right eyebrow), if the scales allowed scoring only for the right side, considering the participants were right-handed. Therefore, for ESRS, rigidity for the right upper limb, which affects speed of hand movement execution, and expressive automatic movement disorders were adopted to assess hand and facial bradykinesia.

#### For Assessing Dyskinesia: AIMS

The AIMS is a clinical scale with seven items for assessing dyskinesia in the upper face (including eyebrows), lips, the jaw, the tongue, upper extremities, lower extremities, and the trunk (32). Each item is scored based on a five-point Likert scale from zero (none) to four (severe). Because this study focused on movements of hands and eyebrows, *upper extremities* and *the upper face* in AIMS were adopted to assess hand and facial dyskinesia.

#### The Interrater Reliability of the Raters

The research personnel who administered the ESRS and AIMS received intensive training from an experienced psychiatrist (the second author) and a researcher with expertise in psychosis-related movement disorders (the first author) through video watching and actual practice. The interrater reliability of the research personnel and the authors was checked: The intraclass correlation coefficient was 0.89 for the hypokinesia factor of ESRS and 0.90 for AIMS. The research personnel was blinded to the research hypotheses.

### The Instrumental Measure of Bradykinesia and Dyskinesia: The VICON Motion Capture System

An eight-camera optical motion capture system (VICON T160; Oxford Metrics Inc., Oxford, UK) was used to quantify the movement speed and quality on the right-hand task and the facial task in participants. The optical cameras generate infrared light to illuminate reflective markers (6.3 mm in diameter for the hand task and 4 mm in diameter for the facial task) attached to the participant's body and capture three-dimensional trajectories of the markers when the subject executes movement tasks. The movements were captured at 120 Hz. The captured data were processed using the VICON Nexus software and further analyzed using the MATLAB R2017a (MathWorks, Natick, MA, USA) to calculate kinematic variables, which reflect bradykinesia and dyskinesia. In order to prevent multiple testing, this study only analyzed movements of the right hand and the right face (the right eyebrow).

#### The Right-Hand Task

**Figure 1:**
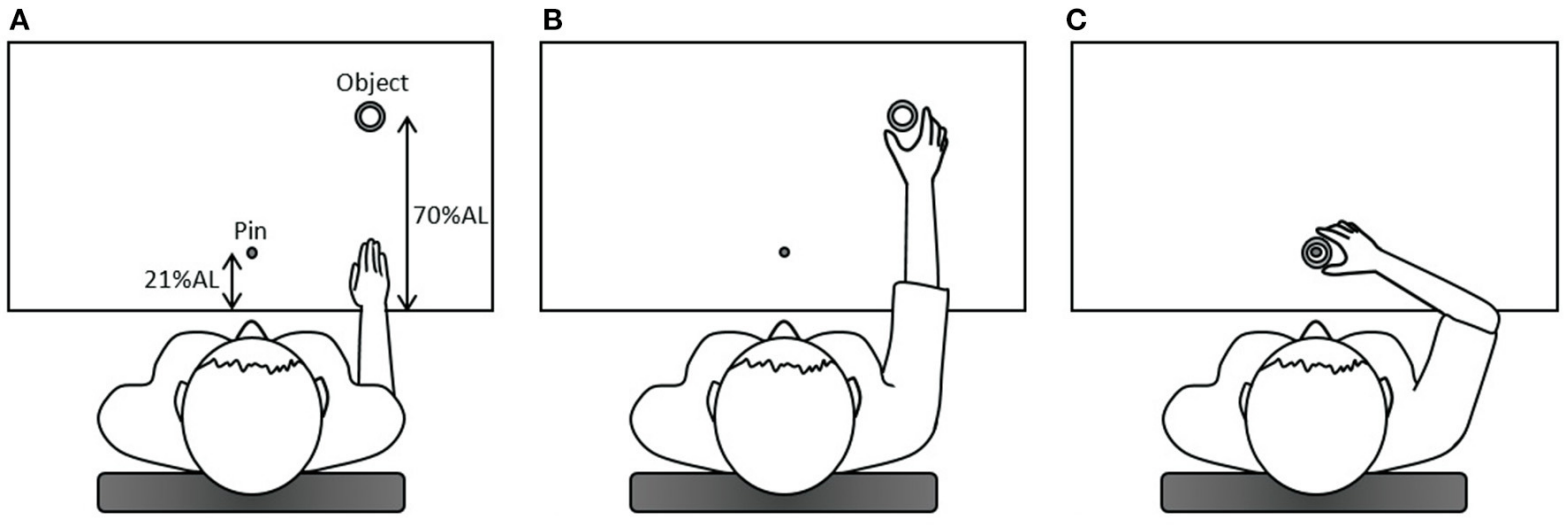
The diagram of the right-hand task. **(A)** In the beginning, the right hand was placed at the starting position; **(B)** upon hearing the starting signal, the participant was required to use the thumb and index finger of the right hand to reach for and grasp the object (the reach-to-grasp movement); **(C)** subsequently, the participant placed the object to the pin, which was the end target. AL, arm length.

**Figure 2:**
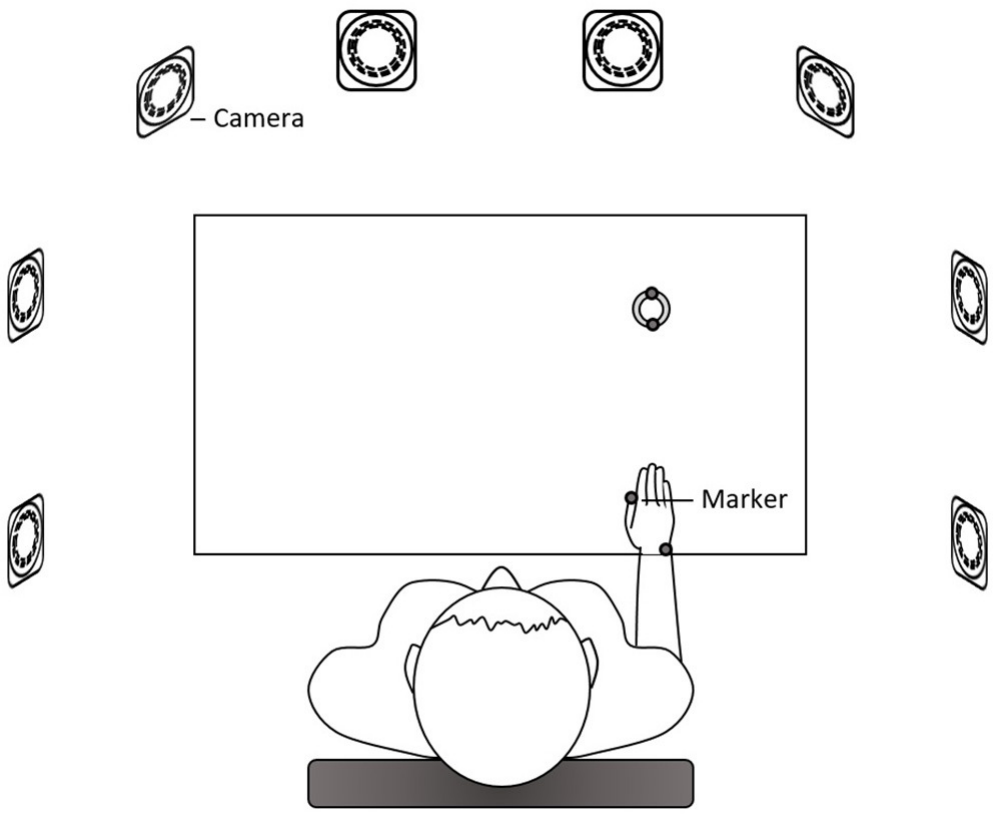
The setup of the right-hand measurement. Eight cameras surrounded the table. Reflective markers were placed on the ulnar styloid process (representing the wrist) and the thumbnail of the right hand and on the object.

The participant sat in front of the table with their trunk harnessed to the chair back to prevent possible trunk movements during the task. The table height was adjusted to the level of the participant's elbow. The right hand was placed at the starting position at a distance of 25 cm away from the midline at the edge of the table in front of the right shoulder. One cylindrical hollow object (outer diameter: 6 cm; inner diameter: 4.4 cm; 1.5 cm high) was placed in front of the starting position at a distance of 70% of the participant's arm length, which was from the axilla to the distal wrist crease (20, 21). A pin (the top was 10 cm high from the table) as the end target was mounted on a base and placed in front of the participant's midline at a distance of 21% of the arm length. Upon hearing the starting signal, the participant was required to use the thumb and index finger of the right hand to reach for and grasp the object and place the object to the pin as quickly as possible (Figure 1). After a practice trial, three data-producing trials were needed. Between each trial, the participant was provided with a short break to allow the hand to leave the table and to prevent fatigue. We analyzed only the reach-to-grasp movement. Two reflective markers were placed on the ulnar styloid process (representing the wrist) and the thumbnail of the right hand and additional two markers on the object (Figure 2). This right-hand task was adapted based on the tasks in previous studies that detected movement abnormalities in schizophrenia patients (25–27).

#### The Facial Task

The participant was seated and required to show blank facial expression in the beginning. Upon hearing the starting signal, the participant needed to make facial expression of surprise to the maximal level as quickly as possible and sustain the highest expressiveness for 1 s. Accuracy of the facial expression was checked by the research personnel via visual inspection. After a practice trial, three data-producing trials were needed. Between each trial, the participant was provided with a short break. A reflective marker was attached to the medial side of the participant's right eyebrow. To exclude influences of head movements on tracking the eyebrow movement, three additional reference markers were placed on the nose tip and the zygomatic process of the temporal bone on both sides of the head, which was used in previous research measuring facial kinematics (24). The facial task used in this study was based on the design of earlier research that detected facial movement problems in patients with impaired basal ganglia (24).

#### Definitions of Kinematic Variables Detecting Bradykinesia and Dyskinesia

For the right-hand task, timing of the movement onset was defined as when the velocity of the wrist marker reached 5% of its peak velocity (20, 22, 23, 25–27). Timing of the movement end was defined as when the velocity of the thumb marker decreased to 0 mm/s and the aimed object kept stationary (25–27). For the facial task, timing of the movement onset and end was defined as when the velocity of the eyebrow marker reached 5% of its peak velocity (20, 22, 23, 25–27) and when its velocity dropped to 5% of its peak velocity (20, 23), respectively.

Bradykinesia was detected using normalized movement time (nMT) (20, 21), which was the interval between the movement onset timing and end timing divided (normalized) by the participant's arm length for the hand movement or divided by the movement displacement of the eyebrow marker for the facial movement. The movement time was normalized because the hand reaching distance and the eyebrow movement displacement varied among participants (20, 21). The nMT reflected movement speed (20, 21): a larger nMT meant a slower movement and more severe bradykinesia.

**Figure 3:**
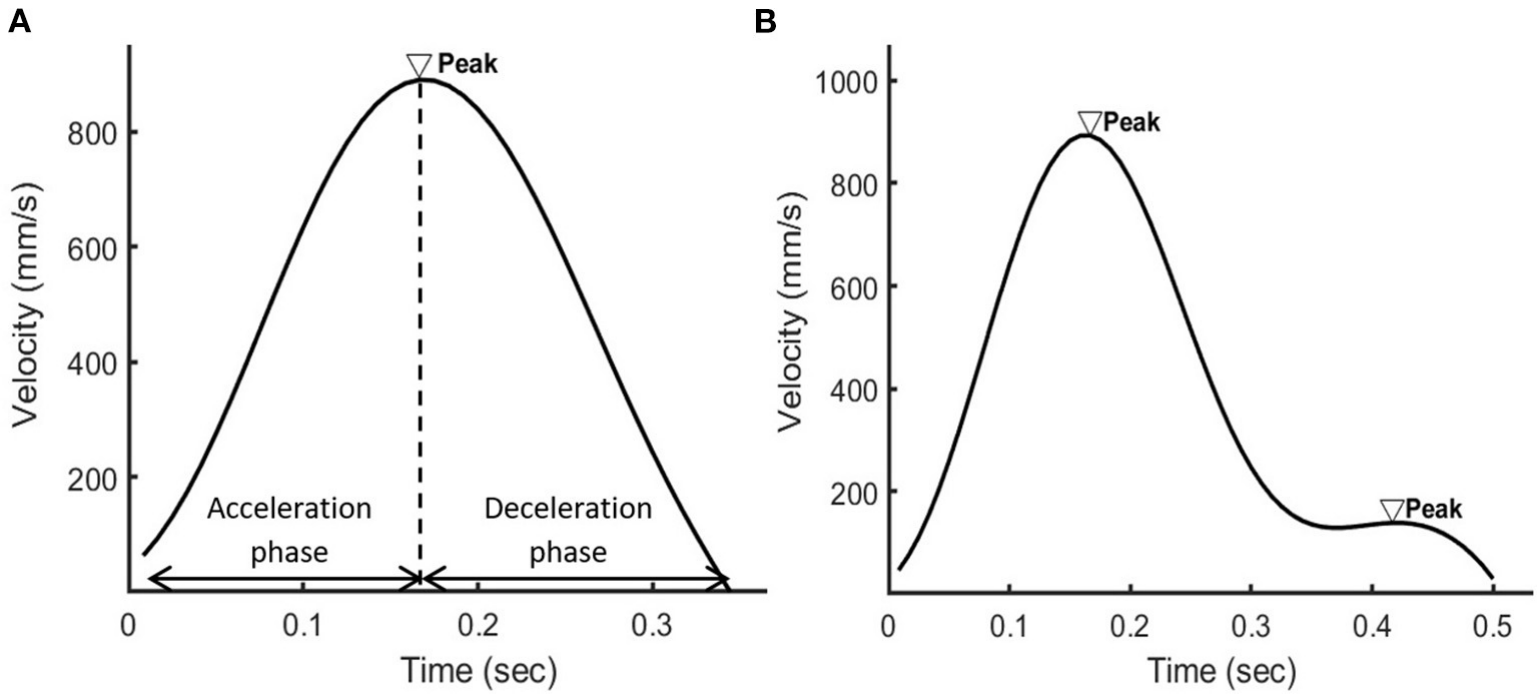
The velocity profile of the wrist marker when **(A)** a healthy person or **(B)** a patient with schizophrenia used the right hand to reach for the object. In the velocity profile of the healthy person, there were one acceleration phase and one deceleration phase in the velocity profile and thus one peak. In the velocity profile of the patient, there were two peaks.

Dyskinesia, involving irregular muscle contraction and thus dysfluent movements (4), was detected using the normalized number of movement units (nNMU) (20, 21). A smooth reaching or eyebrow-raising movement generates one acceleration phase and one deceleration phase, which form a peak in the velocity profile (Figure 3). The nNMU meant the number of peaks in the velocity profile divided (normalized) by the participant's arm length for the hand movement or divided by the movement displacement of the eyebrow marker for the facial movement. The nNMU reflected movement smoothness (20, 21): a larger nNMU meant a less smooth movement and more severe dyskinesia.

### Statistical Analysis

In order to calculate the test–retest reliability of the motion capture system in terms of kinematic variables (hand nMT, facial nMT, hand nNMU, and facial nNMU), the first two data-producing trials of the hand and facial tasks were used. The reason for using the first two trials, not involving the third trial here, was that the first two trials were not or less affected by practice effects of movements, considering that this study collected data of three trials. Pearson or Spearman correlation analysis was conducted according to the Shapiro–Wilk test results of data distribution. The α level (two-sided) was set at 5%. Cohen's standards were adopted for interpreting the correlation magnitude (33): correlation of 0.1 is small/weak, that of 0.3 is moderate, and that of or >0.5 is large/strong.

In order to test group differences in values of kinematic variables (hand nMT, facial nMT, hand nNMU, and facial nNMU) and item scores of ESRS and AIMS, the independent-samples *t*-test or the Mann–Whitney *U* test was used according to the Shapiro–Wilk test results of data distribution. The α level (two-sided) was set at 5%. The value of each kinematic variable was the average of data of the three trials. The effect size *d* of each group difference was calculated (33–35). According to Cohen's suggestions (33), the *d* values 0.2, 0.5, and 0.8 or greater reflect small, medium, and large effects, respectively.

## Results

### Characteristics of Participants

A total of 16 schizophrenia patients and 20 age- and gender-matched control subjects met the inclusion criteria and were recruited in this study (Table 1). Patients had lower scores on the Montreal Cognitive Assessment than did control subjects.

**Table 1:** Characteristics of participants.

	**Patients (*n* = 16)**	**Healthy controls (*n* = 20)**	**Group differences^a^**
	**Mean (SD)**	**Mean (SD)**	***p*-value**
Age (years)	32.34 (12.70)	31.87 (12.16)	0.838
Education (years)	14.59 (2.39)	16.75 (3.18)	0.014
MoCA scores	26.75 (1.95)	27.95 (2.14)	0.049
EHI scores	89.38 (11.24)	87.00 (11.29)	0.560
Illness duration after diagnosis (years)	2.27 (0.75)^b^	—	—
Chlorpromazine equivalents (mg/day)	387.86 (186.56)^c^	—	—
PANSS-Positive symptoms	12.63 (2.50)	—	—
PANSS-Negative symptoms	10.38 (2.53)	—	—
PANSS-General psychopathology	25.25 (4.09)	—	—
	**% (n)**	**% (n)**	***p*** **-value** ^d^
Male	50.00 (8)	75.00 (15)	0.121

*MoCA, Montreal Cognitive Assessment; EHI, Edinburgh Handedness Inventory; PANSS, Positive and Negative Syndrome Scale*.

a *Mann–Whitney U-test because data were not normally distributed*.

b *n = 10 due to lack of data of the year when the patient got the diagnosis for one outpatient and five patients from community self-help groups*.

c *n = 11 due to lack of medication data for five patients from community self-help groups*.

d *χ^2^ Test*.

### Test–Retest Correlation of Kinematic Variables

Because kinematic data of the first two data-producing trials were not normally distributed, Spearman correlation coefficients were calculated. Strong test–retest correlation was found for hand and facial nMT and nNMU (Table 2).

**Table 2:** Test–retest correlation of kinematic variables in participants (*N* = 36).

		**Mean (SD)**	**Spearman correlation**	
			**ρ**	***p*-value**
**Hand**				
nMT			0.920	<0.001
	Trial 1	0.0013 (0.0003)		
	Trial 2	0.0013 (0.0003)		
nNMU			0.995	<0.001
	Trial 1	0.0031 (0.0003)		
	Trial 2	0.0032 (0.0007)		
**Face**				
nMT			0.744	<0.001
	Trial 1	0.0871 (0.1072)		
	Trial 2	0.1444 (0.3184)		
nNMU			0.818	<0.001
	Trial 1	0.3842 (0.5387)		
	Trial 2	0.4907 (0.8542)		

*nMT, normalized movement time; nNMU, normalized number of movement units*.

### Group Differences in Bradykinesia and Dyskinesia

Because the values of kinematic variables and item scores of ESRS and AIMS were not normally distributed, the Mann–Whitney *U*-test was used. Group differences in hand and facial bradykinesia and dyskinesia were not found when we adopted the items of ESRS and AIMS (Table 3), whereas these group differences were found when we adopted the kinematic variables. Patients had larger hand nMT, facial nMT, hand nNMU, and facial nNMU than did control subjects. For group differences in hand and facial bradykinesia, the motion capture system generated larger effect sizes of group differences than did ESRS (the motion capture system: large and large effects, respectively; ESRS: small and medium effects, respectively). Similarly, for group differences in hand and facial dyskinesia, the motion capture system generated larger effect sizes of group differences than did AIMS (the motion capture system: medium and large effects, respectively; AIMS: none and small effects, respectively).

**Table 3:** Group differences in bradykinesia and in dyskinesia detected using clinical measures and instrumental measures.

**Measures**	**Patients** **(*n* = 16)**	**Healthy controls** **(*n* = 20)**	**Mann–Whitney** ***U*****-test**		**Effect size**
	**Mean (SD)**	**Mean (SD)**	**Statistic**	***p*-value**	***d***
**Bradykinesia**					
**The clinical measure (ESRS items)**					
Hand^a^	0.31 (0.70)	0.05 (0.22)	183.00	0.479	0.246
Face^b^	0.56 (0.89)	0.00 (0.00)	220.00	0.058	0.672
**The instrumental measure (nMT)**					
Hand	0.0014 (0.0003)	0.0012 (0.0002)	245.00	0.006	1.011
Face	0.1726 (0.1951)	0.0498 (0.0258)	238.00	0.012	0.909
**Dyskinesia**					
**The clinical measure (AIMS items)**					
Hand^c^	0.94 (0.85)	0.80 (0.83)	174.50	0.648	0.154
Face^d^	1.31 (1.14)	0.80 (0.70)	199.50	0.211	0.429
**The instrumental measure (nNMU)**					
Hand	0.0034 (0.0005)	0.0031 (0.0003)	224.00	0.042	0.722
Face	0.6680 (0.6552)	0.2067 (0.0856)	242.00	0.008	0.966

*ESRS, Extrapyramidal Symptom Rating Scale; nMT, normalized movement time; AIMS, Abnormal Involuntary Movement Scale; nNMU, normalized number of movement units*.

a *The ESRS item was rigidity at the right upper limb*.

b *The ESRS item was expressive automatic movement disorders (facial mask and unintelligible speech)*.

c *The AIMS item was upper extremities*.

d *The AIMS item was the upper face*.

### Additional Analysis: Correlation of Kinematic Variables to Items of ESRS and AIMS

Additional Spearman correlation coefficients (Table 4) were calculated to explore correlation between nMT and the ESRS items (rigidity for the right upper limb and expressive automatic movement disorders) and correlation between nNMU and AIMS items (upper extremities and the upper face). Correlation was found only between facial nMT and the facial item of ESRS (expressive automatic movement disorders) (ρ = 0.378, *p* = 0.023). The relatively small sample size in this study may restrict generalizability and statistical power to find other correlations. Therefore, this additional analysis was conducted only for the exploring purpose.

**Table 4:** Spearman correlation ρ between nMT and the ESRS items and between nNMU and the AIMS items (*N* = 36).

	**The instrumental measure—nMT**	
	**Hand**	**Face**
**The clinical measure—ESRS**		
Hand: Rigidity at the right upper limb	−0.064	—
Face: Expressive automatic movement disorders	—	0.378^*^
	**The instrumental measure—nNMU**	
	**Hand**	**Face**
**The clinical measure—AIMS**		
Hand: Upper extremities	−0.291	—
Face: Upper face	—	−0.031

*nMT, normalized movement time; ESRS, Extrapyramidal Symptom Rating Scale; nNMU, normalized number of movement units; AIMS, Abnormal Involuntary Movement Scale*.

* *p < 0.05*.

## Discussion

The motion capture system had strong test–retest reliability for measuring hand and facial bradykinesia and dyskinesia, supporting the first research hypothesis. In addition, for differences in hand and facial bradykinesia and dyskinesia between schizophrenia patients and healthy people, the motion capture system generated larger effect sizes of the group differences than did the clinical rating scales, supporting the second research hypothesis.

Similar to the previous instrumental measures (17, 18), the motion capture system was reliable in measuring hand bradykinesia and dyskinesia in schizophrenia patients. The system further demonstrated strong reliability as well for measuring facial bradykinesia and dyskinesia, which showed broad applicability of the motion capture system for movement studies in schizophrenia. In the results, the test–retest reliability of the system was weaker, albeit still strong, for measuring facial movement problems than hand movement problems, which may be due to the feature of facial movements *per se*. The sophisticated set of facial muscles enables complex and rich movements of the facial skin, which may cause movement variation across different trials in motion analysis and subsequently reduce associations between movement trials in the test–retest reliability testing. Indeed, earlier research has anticipated lower reliability of mechanical instruments for measuring facial movement problems than hand ones (18).

The motion capture system was more sensitive to detecting differences in bradykinesia and dyskinesia between patients and healthy people than the clinical rating scales, which is consistent with earlier evidence that hand instrumental measures are more sensitive than rating scales (12, 15, 16, 19, 25, 26). This study further showed that the motion capture system was also more sensitive than the rating scales in terms of detecting differences in facial bradykinesia and dyskinesia between patients and healthy people. It is noteworthy that ESRS still generated the medium effect size of the group difference in facial bradykinesia in this study, which may be partly because the facial item of ESRS was designed to assess facial bradykinesia occurring in the whole face, not at one part of the face. Nevertheless, this study showed that even though the motion capture system was used to focus only on the right eyebrow of the participant and measure facial bradykinesia, it still generated a larger effect size of the group difference in facial bradykinesia and was more sensitive than ESRS.

This study had several limitations. First, the sample size was relatively small, which may explain the lack of group differences in hand and facial bradykinesia and dyskinesia assessed using the clinical rating scales. Nevertheless, in the situation of the current sample size, the use of the motion capture system was able to detect the group differences, which supported that the motion capture system was more sensitive than the clinical rating scales in the detection. Future research is suggested to increase the sample size to further examine a correlation between kinematic variables of the motion capture measurement and different items of the clinical rating scales. Second, this study focused on the measurement of hand movements and eyebrow movements, which have been demonstrated to be impaired in patients with schizophrenia or aberrant basal ganglia in earlier studies using motion analysis (24–27). In addition, in order to prevent multiple testing and inflated type I errors, as well as considering that participants were right-handed, this study measured only movements of the right hand and the right eyebrow. Therefore, movement problems occurring in the lower face, including the lips and the jaw, at the other body parts, and on the left side of participants were not measured in this study. Similarly, this study focused only on specific items of the clinical rating scales and not on all items of the scales. In future research, using the motion capture system and the complete clinical rating scales to measure/assess movement problems in the other bodily or facial parts in schizophrenia patients could be considered to explore more topics. For example, it is interesting to examine which bodily or facial parts, not only the wrist and the eyebrow, show kinematic movement problems that are significantly correlated with items of the clinical rating scales. Notably, the motion capture system may not be suitable for measuring tongue movements and thus cannot be used to detect dyskinesia occurring at the tongue. Other instruments, such as load cells (15), together with the motion capture system are required if dyskinesia occurring at the tongue in addition to the face is the research focus. Third, we cannot compare the effect sizes of group differences in this study using the motion capture system with those in literature adopting other hand instruments because different studies recruited different samples of schizophrenia patients. Specifically, when we compare results of different studies, it is difficult to explain whether the larger effect sizes of group differences result from the choice of instruments or a patient sample with more severe movement symptoms. Future research needs to apply different movement instruments to a patient sample and a healthy people sample in one study to compare effect sizes of group differences for different instruments. Last, when collecting data of the test–retest reliability of the motion capture system, this study did not record the time interval between the two movement trials, considering that the two trials were executed within one experimental session, not on different days. Future research may consider separating the first test and the retest of movements by several days or 1 week to examine if the test–retest reliability of the motion capture system differs from the results in this study.

## Conclusions

The major contribution of this study is to demonstrate that the motion capture system, which could be used to measure both bradykinesia and dyskinesia at the body and in the face of the participant, was a reliable, sensitive, and appropriate movement measure for schizophrenia patients. In addition, this study using the motion capture system provided a detailed measuring procedure, which may serve as a reference for future research and clinical practice. To sum up, this study showed that (1) the system was reliable in measuring hand and facial bradykinesia and dyskinesia, and (2) the system was more sensitive than the clinical rating scales in terms of detecting differences in hand and facial bradykinesia and dyskinesia between schizophrenia patients and healthy people. Future research or clinical practice may consider applying the motion capture system to measuring the hand and facial movement problems in schizophrenia patients to gain a deep understanding of the movement issues in schizophrenia. The system may also be applied to measuring subtle movement abnormalities in individuals at risk of psychotic onset to explore the association of hand and facial bradykinesia and dyskinesia to psychosis progression.

## Data Availability Statement

The raw data supporting the conclusions of this article will be made available by the authors, without undue reservation.

## Ethics Statement

The studies involving human participants were reviewed and approved by the Ethical Review Boards of the University (reference number: HSEARS20190322003) and the Hospital (reference number: KC/KE-18-0118/FR-2). The patients/participants provided their written informed consent to participate in this study.

## Author Contributions

S-MW contributed to conceptualization, methodology, data analysis, and writing of the first draft. W-CO contributed to methodology and data analysis. H-MH and L-TH contributed to the task designs of motion analysis, writing of the MATLAB program, and data processing. All authors contributed to manuscript reviewing.

## Funding

This study was partially supported by the Departmental General Research Fund (1-ZE8H) of Hong Kong Polytechnic University.

## Conflict of Interest

The authors declare that the research was conducted in the absence of any commercial or financial relationships that could be construed as a potential conflict of interest.

## Publisher's Note

All claims expressed in this article are solely those of the authors and do not necessarily represent those of their affiliated organizations, or those of the publisher, the editors and the reviewers. Any product that may be evaluated in this article, or claim that may be made by its manufacturer, is not guaranteed or endorsed by the publisher.
